# Supplementary material for: Are carbon emissions trading and green financial instruments synergistic? -Comprehensive quantitative research based on content analysis
Source: PLoS One. 2024 Mar 7;19(3):e0298601. doi: 10.1371/journal.pone.0298601 (PMC10919601; doi:10.1371/journal.pone.0298601)
Supplement: S1 Data — (ZIP) [file pone.0298601.s001.zip › Data/地级市Z_F工作报告.zip!/╜¡╦╒/─╧═¿╩╨/└┤╫╘╣1⁄2╓┌║┼í╛┬φ┐╦╩2╛▌═°í┐_2008.docx]

各位代表：

　　现在，我代表市十二届人民政府向大会作工作报告，请予审议，并请市政协各位委员和其他列席人员提出意见。

　　一、过去五年的工作

　　市十二届人大一次会议以来，我们坚持以邓小平理论和"三个代表"重要思想为指导，认真落实科学发展观，根据省委、省政府对我市"争当全省江北'两个率先'排头兵"的总体定位，按照"依托江海、崛起苏中、融入苏南、接轨上海、走向世界、全面小康"的总体思路和市第十次党代会确定的"建设经济发达、文化繁荣、政治清明、社会和谐、人民安康的新南通"的奋斗目标，紧扣全面小康、全面腾飞主题，在市委的正确领导下，在市人大、市政协的监督和支持下，依靠全市人民，抢抓重要战略机遇期和沿江沿海开发的历史性机遇，开拓奋进，扎实工作，着力推进跨越发展、科学发展、和谐发展，在多年蓄势的基础上，全市进入了又好又快发展的新阶段。

　　2007年全市继续保持了跨越发展的良好态势。全年预计实现GDP2111亿元，按可比价计算增长16.2%，增幅居全省第一；财政总收入300.71亿元，地方一般预算收入127.69亿元，分别比上年增长38.2%和38%，增幅创历史新高；节能减排目标全面实现；全社会固定资产投资1265亿元，增长20.7%；注册外资实际到账31.2亿美元，增长21.4%；社会消费品零售总额733亿元，增长17.8%；城镇居民人均可支配收入16450元，农民人均纯收入6900元，分别增长17%和13%；城镇登记失业率3.2%。2007年各项任务的全面完成，为实现市十二届人大一次会议确定的任期目标画上了圆满的句号。

　　经过五年奋斗，对长远发展有重大影响的基础设施建设和新兴产业培育实现了历史性突破。

　　--大通道建设取得决定性成效。由大桥、公路、铁路、机场、汽渡等组成的综合立体交通网络初步形成，南通正加速向区域性枢纽城市迈进。对融入上海一小时都市圈具有决定意义的苏通大桥全线贯通、即将通车。崇启大桥开工建设的前期工作基本完成，崇海大桥、沪通铁路前期工作取得重大突破。通启高速、沿海高速南通段建成通车，江海高速海安如皋段开工建设，沿江高等级公路全线通车，"一横一纵"的高速公路主骨架和"五横六纵一环"的干线公路网全面建成。宁启铁路宁通段建成通车，铁路南通站始发列车达9对，结束了没有长途客运始发列车的历史。机场航线、航班增加，改造和新建的五大汽渡渡运能力提高50%。

　　--大港口建设实现质的飞跃。以江港能级提升、海港初展雄姿为标志，南通正加速向"江海时代"迈进。沿江泊位功能调整和升级改造取得重大进展，新建、改建5万吨级以上泊位17座（累计25座），接卸超大型船舶能力增强；南通港2006年跻身全国沿海十大亿吨港行列，2007年货物吞吐量1.23亿吨，为2002年的3.28倍。洋口港开发框架全面拉开，黄海大桥工程量完成70%，太阳岛LNG接收站区块围填成功；吕四港2个3.5万吨级散货码头正在加紧建设，沿海深水大港的蓝图即将成为现实。

　　--大产业发展呈现强劲集聚态势。以装备制造业等为代表的新兴产业快速发展，南通正由传统轻纺工业名城加速向长三角北翼先进制造业和基础产业基地迈进。装备制造、电子信息、石油化工、电力能源、现代纺织服装、轻工食品等六大产业占全部工业的比重接近90%，新材料、新能源等新型产业集群化态势强化；造船、制纸、电力装备、港口机械等临港产业加速集聚。

　　--大城市框架迅速拉开。"一主三副"的大城市格局初步确立，南通正向江海交汇的现代化国际港口城市、国内一流的宜居创业城市迈进。市区"五环十四射"路网正在形成，覆盖面积约为2002年建成区的4倍，主城区功能日趋完善；新城区雏形显现，北翼新城框架初步形成，市区常住人口超过100万人，各县（市）城区人口均突破18万人，全市城市化率49.7%、五年提高10.7个百分点，中心城市要素集聚和辐射带动能力不断增强。连续成功举办系列重大活动，"中国近代第一城"开放、包容、文明的新形象充分展示，城市知名度和美誉度持续提升。

　　经过五年奋斗，经济发展实现了全面崛起苏中、初步融入苏南的跨越。

　　--综合实力在持续跨越中快速攀升。GDP增速2004年超过全省平均水平，2005年起连年保持全省领先；总量2003年超过1000亿元，2007年突破2000亿元，在全国大中城市排名五年内由第35位前移至第26位，城市综合竞争力明显增强。五年来，GDP增长1.44倍；财政总收入、地方一般预算收入分别增长2.44倍和2.3倍；进出口总额增长2.16倍。县域经济综合实力快速提升，南三市多项总量指标分别在全市保持领先，北三县（市）多项主要指标增幅分别跃居全市前列，南北差距总体逐步缩小。省定全面小康的18项25个指标有17个达标。

　　--发展活力在外资民资双重推动下显着增强。开放型经济重返全省第一方阵，注册外资实际到账五年累计90.6亿美元，2007年比2002年增长12倍，跃居全省第二、跻身全国十强。全民创业态势强劲，新增私营企业、个体工商户数和个体工商户总数连续四年列全省首位；成为省内第二个、全国第六个私营企业超10万家的城市；私营企业注册资本突破2000亿元，在全省排名五年内由第5位前移至第2位。建筑企业成功实现体制转型，建筑业市场竞争力居全国前列。

　　--经济质态在发展方式转变中明显优化。五年来，三次产业结构由16.3∶51.6∶32.1优化为8.2∶56.7∶35.1，财政总收入占GDP比重由10.1%提高到14.2%，工业经济效益综合指数由132.2提高到240。第一产业内部结构继续优化，增加值五年增长23.6%，高效农业面积和占比均居全省各市前列。五年来，高新技术产业产值增长7.3倍，占规模以上工业比重由13.2%提高到23.8%。在产能翻番的情况下，万元GDP综合能耗下降一成多；主要污染物COD、SO2排放强度分别下降48%和46%，近两年COD和SO2排放总量分别下降6.2%和11.8%。

　　经过五年奋斗，社会建设正呈现出全面进步、和谐奋进的生动局面。

　　--公共服务和社会管理水平在制度创新中不断提升。建立财政投入向公共服务领域倾斜的支出保障制度，五年来财政公共服务投入232亿元，占全部财政支出的47.9%。在全省率先对城乡所有义务教育阶段学生免收学杂费，率先建立城镇居民、农村居民、专业渔民低保制度和被征地农民基本生活保障制度，率先建立计划生育贫困家庭救助、低保边缘群体临时救助等制度。城乡社会保险覆盖面不断扩大。农村社区卫生服务体系、城镇住房保障体系、城乡社会救助体系基本形成。突发公共事件应急机制、食品安全动态监测机制初步建立。在全国首创并不断优化社会矛盾纠纷大调解机制，平安南通建设扎实有效，再获"全国社会治安综合治理优秀市"称号，在首届中国最安全城市评选中，我市位居全国地级市首位。继续蝉联"全国双拥模范城"称号。

　　--人民群众在又好又快发展中得到更多实惠。五年来，城镇居民人均可支配收入、农民人均纯收入分别增长90%、67%；城乡居民储蓄存款余额、社会消费品零售总额分别增长96%、108%；累计提供城镇就业岗位110万个，城镇登记失业率持续低于全国、全省平均水平。2007年底农村劳动力转移总量216万人，全省领先。城镇人均住房建筑面积30.22m2，农村人均钢筋、砖木结构住房面积50.4m2，比2002年底分别增加6.3m2、5.1m2。

　　--全市上下在大发展中形成了政通人和、心齐气顺、风正劲足的良好氛围。"创业创新创优"的新时代江苏精神和"包容会通、敢为人先"的新时期南通精神得到弘扬，社会各阶层和全体建设者积极性持续高涨，社会各方面和衷共济，全市上下始终保持了昂扬向上、团结拼搏、奋发实干的精神状态。

　　这是南通人民的光荣与自豪，也是南通人民宝贵的精神财富，必将激励南通人民在"全面达小康、建设新南通"征程中追求新的更加美好的理想和目标！

　　五年来，我们主要抓了以下几个方面工作：

　　（一）坚持"两个率先"，着力推进跨越发展。

　　以沿江沿海开发为主战场，不断拓展发展新空间。大力实施沿江开发、江海联动战略，五年共完成150项重点工作。推动江海岸线高效利用、港口联动开发。制定并实施《南通市沿江开发详细规划》、《南通港总体规划》等7项规划。整合整治开发沿江深水和中深水岸线23.34公里，新增万吨级以上泊位18座。洋口港、吕四港开发实质性启动并取得重大突破。推动临港产业集群发展。新兴支柱产业快速崛起，沿江先进制造业走廊基本形成。造船完工量274万载重吨，为2002年的5.5倍；电子信息产业增加值五年增长5.1倍。占全市面积30%左右的沿江前沿区域实现了全市近60%的GDP、60%以上的工业销售收入、65%左右的财政收入，比2002年分别提高6个、8个、5个百分点。沿海电力及新能源等基础产业发展态势良好，已建及在建4个风电项目装机总容量45万千瓦。科学统筹沿江沿海产业布局，各县（市）临港临海产业园区要素集聚功能不断增强。

　　以改革开放为主动力，不断激发发展新活力。积极创新大发展的体制机制。国有企业改革实现决战决胜，国有经济布局调整基本完成，市级国有资产管理、监督和营运体系初步建立；事业单位分类改革走在全国前列；行业协会、商会作用进一步强化；投融资体制改革深入推进，新增境内外上市企业7家。积极构建"外事、外资、外贸、外经、外智、外宣"统筹的大开放格局。持续推动利用外资"撑杆跳"，注册外资实际到账年均增长67.1%，新批项目平均单体规模1040万美元、为2002年的7.8倍，进出口总额127.8亿美元、年均增长26.2%，外经合作连年保持全省领先；口岸通关效率进一步提高；全市省级以上开发区GDP占全市比重提高23.4个百分点，南通经济技术开发区GDP增长2.87倍；对外交流与合作不断扩大；引进国外智力工作全省领先。积极争创江苏民营经济第一大市。大力推动全民创业，积极实施"名企名品名人"工程，民资投入占全社会投入比重达75%，民营经济增加值占GDP比重达47%，中国名牌产品总数达26个、跃居全省第三；建筑业施工产值超过1300亿元，为2002年的3.4倍。积极引进国有资本投入。石油、电力、造船、钢铁、化工、重装备等行业的16家中央国有大型企业的重大项目纷纷落户南通，五年投入210亿元。

　　以重大项目为主抓手，不断形成发展新支撑。主攻牵动产业发展的重大项目，五年共实施市级重点建设项目255个、完成投资超过1300亿元，新开工亿元以上项目530个、完成投资894亿元。全社会固定资产投资五年累计4183亿元、增长3.1倍。投资超100亿元的王子制纸、江苏LNG，投资50亿元左右的中远川崎二期、熔盛造船以及19个投资超10亿元的产业项目相继开工建设。

　　（二）坚持统筹协调，着力推进科学发展。

　　突出加快新型工业化，推动经济结构优化升级。大力实施"以工兴市"第一方略，规模以上工业增加值增幅连续47个月超全省平均水平，总量前移至全省第四；工业增加值突破1000亿元、占GDP比重由43%提高到47.8%。加速发展先进制造业和基础产业，规模以上工业增加值中重工业占55%，比2002年提高10个百分点；电子信息、机械、电力、船舶、冶金、化工医药行业增加值占规模以上工业的比重超过50%。着力扶持大企业（集团）发展，年销售收入超10亿元工业企业39家，为2002年的7.8倍。大力推动服务业发展，重点发展物流、金融等生产性服务业，现代服务业增加值占服务业的比重五年提高5.2个百分点。全社会物流总量2.3亿吨，五年增长1.5倍。年末金融机构本外币贷款余额1497.96亿元，为2002年末的2.8倍。国家4A级、3A级景区五年分别增加3个和11个，被评为"中国优秀旅游城市"。

　　突出科教优先、环保优先、节约优先，推动可持续发展。深入实施科教兴市、人才强市战略，跻身全国科技进步先进市和人事人才工作先进行列，在全国率先实现创建全国科普示范县（市）区"满堂红"。2007年全社会研发投入27.65亿元，为2002年的5.9倍。南通高新技术创业中心成功创建国家级孵化器，产学研相结合的技术创新体系初步建立。不断加大环境保护力度，全社会环保投入五年累计197亿元。污水日处理能力由7.8万吨增加到48.8万吨，其中市区由5.3万吨增加到25.5万吨，市区生活污水处理率75.6%、提高47个百分点。提高环境准入门槛，积极淘汰落后产能，对化工企业进行专项整治，关闭小化工企业115家。启动生态市创建，获"保护臭氧层示范市"、"全国水环境治理优秀范例城市"称号，六县（市）全部建成国家级生态示范区。严格海域使用管理，海洋环境保护和生态建设得到加强。启动实施"1668"行动计划和"双百"工程，资源能源利用水平不断提高。严格耕地保护目标责任，连续16年实现耕地占补平衡。

　　突出区域统筹，推动城乡协调发展。加大统筹城乡发展力度，扎实推进"民富、村美、风气好"的新农村建设。实施农民收入倍增工程，加快发展现代农业。坚持以项目农业助推高效农业规模化发展，提高农业综合生产能力，农业综合开发和农机装备水平显着提高，农产品出口创汇和标准化工作位居全省前列。以税费改革为重点，深化农村改革，农民负担明显下降，村级债务总体下降50%。加强农村基础设施和环境建设，农村五件实事全面完成，新五件实事有序推进；农民集中居住区村庄建设规划全面完成，小康示范村建设取得阶段性成效。按照"拓展规模、完善功能、雕琢个性、提升形象"的总要求，大力推进城市建设。强化规划龙头作用，五年编制规划103项，城乡规划基本全覆盖。加大中心城市建设力度，市区城建投入五年累计超过200亿元。完善市区道路网络，人均道路面积15.5m2，五年新增7m2。实施公交优先方针，公交出行率得到提高。市区日供水能力由50万吨增加到90万吨。电话号码升至8位。大力加强城市管理，着力改善城市环境，濠河综合整治和历史风貌保护工程获中国人居环境范例奖；市区人均公共绿地面积9.8m2，五年增加4.9m2；130多个老小区和67个新村组团实行了集中改造，配套设施进一步完善。大力推进"五城同创"，建成国家环保模范城市、国家卫生城市，创建园林城市通过国家专家组评审，完成国家历史文化名城申报工作，文明城市创建经验成为全国典型。

　　（三）坚持以人为本，着力推进和谐发展。

　　不断完善保障体系。统筹推进城乡养老保险，城镇基本养老保险参保86.5万人，农村养老保险参保120万人，比2002年分别增加26万人、8.61万人。加快城乡医疗保险全覆盖，城镇职工基本医疗保险参保114万人，为2002年的2.3倍；城镇居民医疗保险全面推开，市区参保24万人，覆盖率99%；新型农村合作医疗参保527万人，覆盖率95.9%。努力提高城乡低保水平，在"应保尽保"的基础上，先后3次提高保障标准。着力构建"六管齐下"住房保障体系，廉租房制度、保障性商品房的做法在全国介绍。抓好特殊群体保障，农村五保户供养水平和集中供养率不断提高。

　　大力发展社会事业。优先发展教育事业，国民教育体系进一步完善。扎实推进素质教育，全市中小学全面建成省合格学校，高中教育基本普及，教育质量持续领先。中高职业院校在校生由9万人增加到19万人。南通大学成功组建，办学规模突破3万人。成人教育、幼儿教育、特殊教育不断加强。加快构建和完善新型公共卫生体系和医疗服务体系。城乡公共卫生体系健全率98%，疾病预防控制体系不断健全，重大疾病防治工作取得新进展。扎实推进文化大市和体育强市建设。建成南通博物苑新馆等一批文化设施，环濠河文博馆群基本形成。新增全国重点文物保护单位5处、国家级非物质文化遗产2项。版权保护工作全国领先。以"江海志愿者"、"爱心邮路"、"无红包医院"等为代表，精神文明"南通现象"在全国产生重大影响。体育会展中心顺利建成。以改革创新精神成功承办举办第九届亚洲艺术节、省第十六届运动会、省第五届园艺博览会、世界大城市带发展高层论坛等系列重大活动。全民健身体系基本形成，全国和省级体育后备人才基地建设取得新成绩。人口和计划生育工作保持全国领先，人口连续10年负增长。广播电视事业快速发展，五年新增有线电视用户100万户。依法维护妇女、儿童和青少年权益，老龄事业、残疾人、关心下一代、老区开发、红十字和慈善等工作进一步加强。

　　全面加强社会管理。扎实推进民主法制建设。基层民主得到发展。"四五"普法受到省委、省政府表彰，"五五"普法进展顺利。法治合格县（市、区）和合格乡镇（街道）创建成效显着，法治南通建设走在全省前列。切实维护社会稳定。公众安全感连续五年全省领先，防范和处理邪教工作被评为全国先进。大调解、大防控体系不断完善。大信访格局加速形成，信访总量五年下降24%。全面加强安全生产，各类安全事故起数和死亡人数连年下降。整顿规范市场经济秩序工作和社会信用体系建设不断强化，食品药品安全监管和市场价格监管扎实有效。国家安全、国防动员、民兵预备役、双拥共建、优抚安置、人防等工作取得新成绩，海安角斜红旗民兵团新时期民兵建设经验在全国推广，如东环港女炮排被省委、省政府、省军区命名为"海防模范女炮排"。新闻出版、哲学社会科学、侨务、台湾事务、民族、宗教、地方志、统计、机关事务管理、气象、防震、档案、无线电管理、保密等工作进一步加强。

　　切实维护群众利益。注意从实际出发，量力而行、尽力而为，尽可能解决好与民生相关的现实问题。积极稳妥推进企事业单位改制，认真落实拆迁安置政策，有效保障广大职工和被征地农民的合法权益。着力构建人民来信来访、政府服务热线、市长信箱"三位一体"的诉求表达机制，五年共受理、答复、处理市民建议、求助、投诉等方面的电子邮件近4万件、来电8万多个、来信来访约30万件次，解决了一大批事关群众切身利益的问题。加强专项治理，切实纠正损害群众利益的不正之风。

　　各位代表，过去的五年，我们积极适应经济社会发展的新形势、新要求，不断转变政府职能，政府自身建设得到加强。坚持服务发展、服务群众。深化机关作风建设，大力争创全省最佳办事环境，认真贯彻《行政许可法》，削减行政审批事项，规范行政许可行为，市级行政审批事项减少811项，减幅78.1%；形成并坚持重大项目领导办公会制度，大企业（集团）、小巨人企业会办制度，外资、民营企业会办制度等系列推进制度，有力促进了各类企业的发展和重大项目的推进。坚持为民办实事制度，累计安排实事项目46件81项，各级财政投入75亿元，其中，市本级财政投入24亿元。坚持优化决策、推动落实。五年共实施重点调研课题近200项，90%以上的调研成果得到转化。从基础做起，在重点上突破，在难题化解上下功夫，市政府确定的525项重点工作全面完成。坚持依法行政、廉洁从政。注重政府法制建设，完善监督协调机制，不断规范行政执法行为，全面推开相对集中处罚权工作，建立行政首长出庭应诉制度，依法保障公民的知情权、参与权和监督权。认真贯彻市人大及其常委会的决议、决定，及时主动报告工作，自觉接受市人大的法律监督和工作监督；主动接受市政协的民主监督及社会各界的日常监督。五年共办理人大议案和政协建议案各10件，办理人大代表建议、批评、意见1644件和政协委员提案2772件，代表、委员满意基本满意率逐年提高，2007年分别达98.1%、97.3%。认真执行党风廉政建设情况报告、述职述廉等制度，严格政府采购制度，全面实行工程建设招投标、经营性土地使用权招拍挂出让制度，试行政府投资项目代建制，不断强化审计监督。严肃查处违法违纪违规行为。

　　各位代表，回顾五年的工作，我们深感成绩来之不易。这一切，归功于省委、省政府和市委的正确领导，得益于历届领导班子打下的坚实基础；这一切，是全市人民齐心协力、拼搏奉献的结果，也离不开所有海内外朋友的关心、支持和帮助。此时此刻，我和市政府全体同志心存感激、充满敬意。请允许我代表市人民政府，向广大干部群众，向人大代表、政协委员和离退休老同志，向各民主党派、工商联、各人民团体和各界人士，向人民解放军、武警驻通部队官兵、公安干警和部、省驻通单位，向一切关心和支持南通发展的海内外朋友，致以崇高的敬礼和衷心的感谢！

　　各位代表，我们清醒地看到，我市经济社会发展和政府工作存在的问题仍不容忽视。一是人均GDP、人均财政收入等仍然低于全省平均水平。二是产业层次仍然不高。第三产业比重还偏低，结构性矛盾仍比较突出。自主创新能力不强，全社会研发投入占GDP比重、高新技术产业产值占规模以上工业比重均低于全省平均水平。三是发展方式有待进一步转变。节能降耗、污染减排的任务还很艰巨，生态环境有待进一步改善。四是农民持续增收难度加大，农村社会保障体系建设还相对薄弱。五是社会建设有待进一步加强。城乡居民就业结构性压力尚未得到根本缓解，部分弱势群体生活还比较困难，医疗、教育和城市交通、住房等方面还存在群众不满意的问题。六是政府自身建设存在薄弱环节。有些部门职能转变滞后，社会管理和公共服务需要继续加强。对此，必须高度重视，认真加以解决。

　　二、今后五年的目标任务

　　今后五年是南通"全面达小康、建设新南通"的决胜期，也是基本实现现代化的起步期，将由"第一个率先"向"第二个率先"迈进。南通已进入一个全面腾飞的黄金发展期，按照省委、省政府对南通提出的"建设江苏新的增长极"的要求，下届政府工作的指导思想建议为：高举中国特色社会主义伟大旗帜，以邓小平理论和"三个代表"重要思想为指导，深入贯彻落实科学发展观，根据党的十七大提出的实现全面建设小康社会奋斗目标的新要求和省、市党代会精神，继续遵循"依托江海、崛起苏中、融入苏南、接轨上海、走向世界、全面小康"的总体思路，坚持跨越发展、科学发展、和谐发展，着力推进改革开放，着力转变发展方式，着力改善人民生活，加快建设经济发达、文化繁荣、政治清明、社会和谐、生态文明、人民安康的新南通，全面建成老百姓认可的更高水平小康社会，奋力开启向基本现代化迈进的新征程。

　　今后五年的奋斗目标建议为：

　　--全面小康建设的水平更高。力争人均GDP等主要均量指标达到或高于全省平均水平，部分均量指标赶上苏南。到2012年，GDP和人均GDP均比2007年翻一番以上，分别突破4500亿元、60000元，经济总量在全国大中城市排名前移2位以上；一般预算收入突破350亿元、年递增20%以上，财政总收入占GDP比重超过17%；城镇居民人均可支配收入、农民人均纯收入分别超过28000元和12000元。

　　--全面小康建设的质量更优。2012年二三产业增加值占GDP比重95%左右。自主创新能力显着增强，2012年高新技术产业产值占规模以上工业比重超过30%，全社会研发投入占GDP比重超过1.8%。全市总体上进入环境优化发展阶段，2012年万元GDP能耗降至0.66吨标煤以下，主要污染物COD和SO2排放总量在2010年比2005年分别削减14.7%和29.5%的基础上继续下降，环境质量综合指数超过85分。区域协调发展机制进一步形成，城乡差距、南北差距不断缩小，2012年城市化率力争达60%。

　　--全面小康建设的进程更快。确保2010年、力争2009年在全省江北率先达到省定全面小康指标值；2010年以县（市）为单位达到省定全面小康指标值，成为全省江北第一个小康县（市）群。同时，不断巩固以市和以县（市）为单位的全面小康成果，加快以乡镇为单位建成全面小康社会，为在下一个十年实现"第二个率先"目标打好基础、争得主动。

　　--全面小康建设的领域更全。经济、政治、文化、社会建设"四位一体"同步推进，基层民主制度更加完善，政治、经济和社会生活纳入法治化轨道，群众权益和公平正义得到较好保障，公众安全感进一步增强；社会主义核心价值观深入人心，全民文明素质不断提升，文化强市建设取得重大进展，覆盖城乡的公共文化服务体系基本形成，建成一批新的惠民利民的文化标志性工程，文化软实力显着增强；社会事业实现更大发展，科教人才优势更好发挥，教育现代化基本实现，2012年高中阶段教育毛入学率和高等教育毛入学率分别超过95%和50%。

　　--全面小康建设的成效更实。城乡居民共享经济社会发展成果，生活质量和生活环境显着改善，大多数家庭达到全面小康生活水平，消除绝对贫困现象。市场物价基本稳定，社会就业更加充分。覆盖城乡居民的社会保障体系基本建立，城镇低收入家庭住房保障制度建设全国领先，人人享有基本生活保障和基本医疗卫生服务，2012年城镇养老、医疗、失业保险覆盖率和新型农村合作医疗覆盖率均达99%。

　　实现上述目标，需要按照科学发展观的要求，突出富民优先、科教优先、环保优先、节约优先，着重把握以下几点：

　　（一）坚持解放思想，更加注重走具有南通特色的又好又快发展之路。坚持实事求是、一切从实际出发，自觉把上级精神与南通实际结合好，主动顺应沪苏通"金三角"发展趋势，抢抓区域枢纽地位加速形成带来的历史机遇，放大靠江靠海靠上海、连通苏北的独特优势，扬长避短，开拓奋进，加速把阶段性优势转变为长期性优势、潜在优势转变为现实优势、局部优势转变为整体优势、现实优势转变为竞争优势。

　　（二）坚持改革开放，更加注重调动一切积极因素。拓展改革开放的广度和深度，构建有利于推动科学发展、促进社会和谐的体制机制。以"包容会通、敢为人先"的新时期南通精神激励斗志，以"创业创新创优"促"企业增效、群众增收、财政增长"，以正确处理和及时疏导社会矛盾提升发展合力，巩固政通人和、同舟共济建设全面小康社会的生动局面。

　　（三）坚持统筹协调，更加注重转变经济发展方式。坚持统筹兼顾的根本方法，更好地统筹城乡、区域、经济社会、人与自然和内外协调发展，增创跨越发展、科学发展、和谐发展的特色优势。坚持好字优先，增强发展的协调性，推动经济增长由主要依靠投资、出口拉动向依靠消费、投资、出口协调拉动转变，由主要依靠第二产业带动向依靠三次产业协同带动转变，由主要依靠增加物质资源消耗向主要依靠科技进步、劳动者素质提高、管理创新转变，加快建设资源节约型、环境友好型社会。

　　（四）坚持以人为本，更加注重改善民生。把改善民生作为构建和谐社会的首要任务，以实现全体市民学有所教、劳有所得、病有所医、老有所养、住有所居为重点，着力增强改善民生的普惠性，着力完善保障民生的制度性安排，着力解决影响民生的突出问题，加强社会建设和管理，大力推进基本公共服务均等化，普遍提高人民生活质量，更多改善城乡低收入群众尤其是困难群众的基本生活，让发展成果惠及更多的老百姓。

　　各位代表，经过五年的努力，南通将率先在江苏江北建成更高水平小康社会，并为基本实现现代化奠定坚实基础；经过五年的努力，南通将取得建设"北上海"的阶段性突破，在构建沪苏通"小金三角"中将有更大作为，在建设江苏新的增长极中将有更大贡献，基本建成融入上海一小时都市圈的区域枢纽城市、江苏江海交汇的现代化国际港口城市、长三角北翼经济中心和国内一流宜居创业城市，全面腾飞的新南通将更加充满生机与活力，南通的明天将更加美好！

　　三、2008年工作建议

　　2008年，是全面贯彻落实党的十七大战略部署的第一年，是实施"十一五"规划承上启下的一年，是建设全面小康社会的攻坚年，也是改革开放30周年和奥运盛会的举办年，做好2008年工作意义重大。当前，宏观经济环境不确定因素有所增加，做好今年工作，要进一步增强又好又快发展的信心，也要充分估计前进中的突出问题和潜在隐忧，进一步增强忧患意识，更加积极地抓好机遇、应对挑战、趋利避害、把握主动。根据市委十届四次全会精神，建议今年全市经济社会发展的主要预期目标为：GDP增长15%左右，万元GDP综合能耗下降4%左右，主要污染物COD、SO2排放总量分别削减3.1%和6.6%，地方一般预算收入增长20%以上，全社会固定资产投资增长18%以上，社会消费品零售总额增长15%左右，城镇居民人均可支配收入、农民人均纯收入分别增长11%和10%以上，居民消费价格总水平涨幅不高于全省平均水平，城镇登记失业率控制在3.8%以内，人口自然增长率控制在零以下。

　　实现上述目标，建议重点抓好以下八个方面工作：

　　（一）进一步推动产业结构优化升级，加快经济发展方式新转变。坚持走新型工业化道路，调整优化产业结构，力争二三产业增加值占GDP比重提高1.3个百分点。大力发展先进制造业。调强支柱产业。重点发展装备制造、电子信息、石油化工、电力能源、现代纺织服装、轻工食品等六大支柱产业，加快向产业链高端攀升。鼓励扶持船舶修造与配套、港口机械、节能环保设备等装备制造业加快发展，力争形成2000亿元的产出规模。围绕打造中国乃至世界"船谷"，强势推进船舶产业发展，力争造船完工量达420万载重吨。大力发展电子信息和新能源、新材料、新医药等新兴产业，力争高新技术产业产值达1150亿元，占规模以上工业比重提高1个百分点。调高产业层次。运用信息技术和先进适用技术改造提升传统产业，不断提高产品附加值和市场竞争力。调大企业规模。继续实施企业梯度培育工程，促进生产要素向优势企业集中，培育一批技术领先、拥有自主品牌的大企业、大集团，确保年销售收入超20亿元工商企业达25家，其中，50-100亿元企业4家，超100亿元企业1家。大力发展现代服务业。重点发展现代物流、金融保险、商务科技等生产性服务业，加快建设现代服务业集聚区，力争服务业比重提高1个百分点、生产性服务业增速比服务业快1个百分点。加快港口物流园区、火车站物流中心等物流载体建设，着力推进沿江货主码头整合，大力发展第三方物流。深入落实金融业发展扶持政策，加快现代金融服务体系建设，完善金融服务功能。大力扶持服务外包、软件、会展等产业发展。加大项目建设和市场开发力度，着力发展集江风海韵与文化底蕴于一体的特色旅游。有针对性地实施土地增量供给措施，强化房地产市场监管，稳定住房价格，推动房地产业健康发展。改造提升传统服务业，积极支持家用纺织、电动工具、花卉苗木、建材家装等专业市场建设，继续发展商贸流通、宾馆餐饮等服务业。大力发展现代高效农业。继续争创全省高效农业规模化第一市，力争亩净收益2000元以上农田占比提高5个百分点，"三资"开发农业投入增长30%以上，新建高效农业规模化示范区2个。着力提升农业产业化经营水平，做强55个市级以上龙头企业，扶持48个重点项目，新建10个重点园区和150个合作经济组织。突出抓好循环农业、生态农业，新增无公害农产品30个、绿色食品15个、有机农产品5个。扩大农业政策性保险试点，强化农业资源综合开发，提高农机装备水平，健全农业社会化服务体系，增强农业综合生产能力。加强重大动物疫病防治。大力提高自主创新能力。围绕建设创新型城市，积极培育科技创新主体，引导鼓励企业加大研发投入，争取新增省级以上高新技术企业20家和市级以上企业技术中心15个，工程技术研究中心、重点实验室20个。加强产学研合作，力争培育200家示范企业，实施200个合作项目。继续推进"人才特区"建设，积极引进高层次人才和科技领军人物。扎实推进国家知识产权试点市建设。加快推进南通科技创业社区建设，提升各级高新技术服务中心孵化功能。大力提高土地节约集约和科学利用水平。严格耕地保护制度，继续保持耕地占补平衡。强化计划调控，切实盘活存量土地，进一步完善重点项目用地直供制度，建立节约集约用地评价体系和考核制度。

　　（二）进一步加大统筹力度，加快构建城乡发展一体化新格局。统筹城乡互动发展。着力推进城乡发展规划一体化、经济发展一体化、基础设施一体化、公共服务一体化、就业社保一体化和管理体制一体化，加强县（市）城和临海、滨江新城建设，加快培育重点中心镇，不断完善以中心城市为核心，县（市）城、中心镇、一般镇和村庄（社区）分工合理、层级分明、功能配套的城乡互动发展体系。加强区域发展统筹规划，加快建立市县两级跨区域间的规划协调、对接机制。积极探索以工促农、以城带乡的新路子，促进城乡生产要素良性互动，加快基础设施、公共服务向农村覆盖，推动城市优质社会事业资源进一步向农村延伸。重点推进城乡道路、水利设施等建设和区域供水，促进基础设施共享共用。加强农村公共服务，基本实现村级公共服务中心全覆盖。深入推进"民富、村美、风气好"的新农村建设。巩固完善强农惠农政策，大力发展农村非农产业，继续实施农民收入倍增工程，促进农业持续增效、农民持续增收、农村持续发展。积极稳妥推进农民集中居住区和农村新社区建设，提高基础设施通达配套水平。开展以"六清六建"为重点的农村人居环境建设和综合整治试点工作。继续强化农村集体资产和财务管理，规范"一事一议"筹资筹劳。发展壮大村级集体经济。继续加大对经济薄弱村扶持力度，抓好扶贫和老区开发。着力增强和完善中心城市功能。完成新一轮城市总体规划修编。组织编制市区分区规划、重要历史街区地段保护规划等18项规划。继续拉开市区道路框架，规划建设出城快速通道，不断完善环内路网。加快新城区东拓、南接、西连和中央商务区建设，提升开发区服务配套功能，推进北翼新城建设。进一步提升主城区功能，注重城市设计和风貌保护，积极推进老小区综合整治和危旧房、"城中村"改造，加快公交枢纽站、换乘中心等基础设施建设，努力解决交通拥堵和停车难问题。继续推进狼山风景区整体开发和苏通大桥北桥头区规划建设。重点做好唐闸地区、寺街历史街区保护性改造。深化落实市容环卫责任区制度，不断完善市区市容环卫管理考核机制。积极推进数字化城管，提高城市综合管理水平。

　　（三）进一步放大开放领先、改革率先效应，加快构筑体制机制新优势。认真总结改革开放30周年的宝贵经验，不断加快市场化和经济国际化进程。继续巩固开放型经济全省第一方阵地位。注册外资实际到账、外贸进出口总额保持较高发展水平，外经主要指标继续保持全省领先，开发区努力跻身全省中上水平。着力扩大开放领域。引导外资投向现代服务业、高端制造业、现代农业、节能环保等产业领域，不断拓展利用外资新空间。重点突破电子信息、服务外包、金融、物流、会展、中介等领域招商，力争取得较大进展。着力优化开放结构。根据内外资企业所得税并轨的新情况，注重发挥区位、资源比较优势，大力提高临港型、临海型、龙头型、基地型项目比重。确立"不求所在、但求所得"的国民财富观，大力实施"走出去"战略，组织推动企业尤其是民营企业到有市场、有资源的国家和地区参与合作。根据国家外贸政策调整方向，推动加工贸易转型升级，确保外贸出口额突破100亿美元，力争机电产品、高新技术产品出口比重提高2个百分点。着力提高开放质量。提高准入门槛，建立招商选资新机制，更多引进土地集约利用水平高、对地方就业和税收贡献大的项目。实行引进项目和引进创新资源相结合，扩大招才引智，引进跨国公司研发机构和地区总部，促进外资企业本土化。加强开发区功能配套、体制创新和区中园建设，注重培育开发园区的政务、商务、生态环境等综合竞争优势，全面提升创新功能和产出效能。力争南通开发区在全国国家级开发区中排名前移2位，13个省级以上开发区GDP占全市比重提高3个百分点以上。进一步完善"外事、外资、外贸、外经、外智、外宣"统筹的大开放格局。继续争创江苏民营经济第一大市。保持个体工商户数全省第一、私营企业数及私营企业注册资本全省第二的地位。重点打造信息技术、咨询服务等八大公共服务平台，推动民营经济由总量与速度的赶超向质量与效益的赶超转变。做强优势板块，进一步发挥50个重点工业集中区集聚功能，壮大家用纺织品、电动工具等20个特色产业集群，力争重点工业集中区规模企业主营业务收入占全市比重提高2个百分点；着力打造全国一流建筑强市，力争建筑业施工产值超过1500亿元。做大单体规模，力争销售收入亿元以上民营工业企业超过500家，规模以上民营企业增加400家。做多优势品牌，深入实施"名企名品名人"工程，争创中国名牌产品5个、驰名商标3件。深入推进重点领域和关键环节的改革。巩固和完善行政审批制度改革、事业单位分类改革、市区两级财权和事权管理体制改革等成果。推进国有资产监管全覆盖，确保国有资产保值增值。完善公共财政体系，提升公共财政保障水平。深化投融资体制改革，继续试行政府投资项目代建制。深化农村综合改革，坚持农村基本经营制度，加快建立市场化的土地承包经营权流转制度，鼓励农村土地集约经营，继续推进集体建设用地使用制度改革。加快现代市场体系建设，进一步发展壮大土地、产权、技术、人力资源等要素市场，积极培育资本市场，力争新增上市公司4家以上。探索绩效管理新形式，促进机关职能转变。探索社会公益型事业单位"管办分离"，加快推进公用事业领域"管养建分离"。深入推进接轨上海。加快基础设施的全面对接，进一步推动港航、交通、信息、金融、旅游等方面的共建共享，进一步加强与上海的产业合作。

　　（四）进一步抢抓大桥通车、海港通航机遇，加快拓展江海开发新空间。按照省委、省政府提出的"苏中及沿海地区要充分利用苏通大桥开通的机遇，着力形成江海联动、跨江联动发展新格局"的最新要求，紧紧抓住大桥通车、海港通航等重大契机，更大力度实施江海联动开发。加快推进港口建设。进一步加快沿海深水大港开发，洋口港全面完成陆岛通道和太阳岛二期工程，同步推进10万吨级液化气码头、万吨级通用码头建设，确保如期通航；吕四港确保建成2个3.5万吨级散货码头，完成进港航道一期工程。进一步整合提升沿江港口功能，重点推进天生港区、南通港区、任港港区岸线整合以及横港沙、如皋沙群、新开沙及其夹槽整治，加快新通海沙、长江北支和如皋港区岸线开发，力争新增万吨级以上生产性泊位4座，全市港口货物吞吐量达1.4亿吨。强势推进临港临海产业集聚。重点发展以中远川崎、熔盛造船、振华港机、二重装备等为代表的装备制造业，以王子制纸为代表的纸制品业，以如东风电等为代表的电力能源业，以南通宝日制钢合金钢、上海二钢线材为代表的钢制品业，以沙钢物流为代表的现代物流业。大力发展海洋经济。全力推进120个市级重点建设项目和60个市级重大储备项目。继续推进重大基础设施建设。做好苏通大桥通车准备工作。开工建设崇启大桥、崇海大桥。推进江海高速海安如皋段、204国道南通段扩建、苏334线复线等续建工程。力争沪通铁路年内开工，加快推进洋口港铁路建设，积极做好沪通城际轨道交通、宁启铁路复线电气化改造、如皋港铁路等前期工作。推动兴东机场升级改造。

　　（五）进一步加大节能减排力度，努力推动生态文明建设新突破。发挥政府主导作用，强化企业社会责任，深入开展节能减排全民行动，建立健全有效的激励和约束机制，确保完成节能减排目标，进一步减少经济发展的资源环境代价。打好节能减排攻坚战。推进工程节能减排，实施节能技改、循环经济、清洁生产和资源综合利用项目200个；重点推进日处理能力２万吨以上的15个污水处理项目建设，力争市区及各县（市）城生活污水集中处理率达80%、乡镇生活污水集中处理率达30%；加快实施华能电厂等脱硫工程。推进结构节能减排，深入开展化工行业整治，提高各类化工集聚区污染集中处理能力，加快姚港化工区重点三类企业搬迁改造，在确保提前完成省定关闭小化工企业任务的同时，对市区39家沿江化工企业实施专项整治；突出抓好冶金、化工、电力、纺织、建材五大重点耗能行业的节能改造，大力推进墙体材料革新和建筑节能；实行总量控制与项目限批挂钩，完不成减排任务的区域，一律不得新上项目。推进监管节能减排，进一步完善执法体系，制定并实行节能减排统计、监测和考核的具体方案和办法，强化目标责任制，严把项目准入关，严格实行节能减排一票否决制和问责制；加快推进通海片区钢丝绳行业整治，开工建设开发区热处理中心和环境基础设施，年内确保完成50%的工程量；根治观音山地区印染行业污染源，基本完成主要河道清淤整治；对100家重点耗能企业、100家重点污染企业实施重点监管，特别是对年排SO2超1000吨的9家电厂和年排COD超100吨的65家企业落实减排监管措施。大力推进生态市创建。以开展多种形式的创建活动为抓手，加强环境建设和生态修复，力争水域功能区水质达标、环境质量综合指数80分以上。加快环境优美乡镇、生态村和绿色单位、绿色家庭创建进程，力争建成国家环境保护模范城市群。着力做好水、大气、土壤等污染防治工作，切实加强饮用水源地环境保护。加强海洋生态环境监管，推进生态海洋建设。提升城市绿化水平，力争绿化覆盖率达42%。加快沿江、沿海、沿河、沿路绿化带建设，力争林木覆盖率达18%以上。积极改善农村生态环境，加强面源污染综合治理，全面实行生活垃圾集中处理。

　　（六）进一步加强社会建设，努力满足人民群众改善民生新期待。办好教育事业。加大财政投入，规范教育收费，全面化解农村义务教育债务，保障经济困难家庭、进城务工人员子女平等接受义务教育。扎实推进区域教育现代化，力争2个县（市、区）达标。深入推进素质教育，提升九年义务教育质量，巩固普及高中阶段教育成果。扩大优质教育资源覆盖面，推动义务教育均衡发展。建设一批公共平台、实训基地，积极支持职业院校改扩建，重点发展与地方经济联系紧密的专业，提高职业院校人才培养的适用性。统筹发展成人教育、幼儿教育和特殊教育。继续支持南通大学加快新校区建设。办好卫生事业。进一步加强公共卫生体系建设，提高城乡公共卫生服务效能和突发公共卫生事件应急处置能力。优化医疗资源配置，尽快发挥综合性医院改扩建效应，促进市区老城区优质医疗资源向港闸区、新城区、开发区延伸，全面提升医疗服务水平。加强镇村、社区和经济薄弱地区卫生机构的基础设施建设，全面建成覆盖城乡的社区卫生服务体系。严格控制医药费用不合理增长，努力减轻群众看病负担。深入创建"平安医院"，巩固"无红包医院"创建成果，构建和谐医患关系。办好城乡就业和社会保障事业。积极实施《就业促进法》，完善城乡公共就业服务体系，鼓励支持自主创业、自谋职业和灵活就业。深入开展对就业困难人员的就业援助，动态消除零就业家庭，实施特困家庭大中专毕业生就业援助工程。认真贯彻《劳动合同法》，规范企业用工行为，普遍实行职工工资集体协商制度，建立企业职工工资正常增长机制和支付保障机制，着力构建和谐劳动关系。完善最低工资标准和城乡最低生活保障标准等正常增长机制。加快推进城乡社会保障制度并轨。不断扩大各类社会保险覆盖面，强化社会保险基金监督管理。扎实开展创建省人口协调发展先进县（市、区）活动，提高出生人口素质。实施计划生育家庭特别扶助制度和市区城市困难群众医疗救助制度，更好发挥江海志愿者作用，鼓励社会力量参与扶贫济困，进一步健全社会救助体系。以解决城镇低收入群众和特殊群体住房困难为重点，进一步完善"六管齐下"的住房保障体系。开工建设市民服务中心。集中财力物力，切实办好12件32项政府为民办实事项目。加强社会管理。进一步加强社区建设，改进物业管理，加快完善专业社工服务中心、外来人口管理服务中心、社会矛盾纠纷调处中心和治安视频监控中心。推动行业协会、商会改革和发展，充分发挥各类社会组织作用。深化平安南通建设。整合社会资源，加强社区警务，提升社会治安大防控水平，严厉打击各种违法犯罪活动，强化防范和处理邪教工作，切实维护社会稳定。畅通群众诉求渠道，完善大信访制度，充分发挥大调解机制作用，有效化解各类社会矛盾。严格安全生产责任制，健全监督体系，坚决防止重特大安全事故发生。切实增强全民国防观念，强化国防后备力量建设，大力支持驻通部队建设。继续做好人防、国家安全、防震减灾工作，加强重大气象灾害应急服务能力建设，全面构建公共安全屏障。深入整顿和规范市场秩序，开展放心消费创建活动，加快公共检测平台建设，建立产品质量和食品药品安全监管长效机制；完善以粮油、猪肉为重点的生活必需品储备体系，全面落实"菜篮子"行政领导负责制，切实做好价格监管和市场调控。更加重视发展老龄和残疾人事业，更好保障妇女儿童合法权益。继续加强哲学社会科学、关心下一代、地方志、统计、档案、无线电管理、保密等工作。

　　（七）进一步提升文化软实力，努力开创文化繁荣新局面。提升社会文明程度。以建设社会主义核心价值体系为引领，大力弘扬精神文明"南通现象"和新时期南通精神。积极实施公民道德建设工程，加强未成年人思想道德建设和青少年思想政治工作，制订实施公民素质提升计划，促进全民综合素质提高。继续推进社会信用体系建设，健全个人和企业联合征信平台。积极开展群众性精神文明创建活动，大力推进文明城市创建，力争建成全国文明城市。强化公共文化服务。建设市图书中心、新闻报业中心等一批文化设施，组织开展文化交流中心前期论证。加快乡镇和社区文化设施建设，力争70%的村建有"文化室"、"农家书屋"。加强文化遗产和非物质文化遗产的保护、开发与利用，传承江海特色优秀文化。大力开展全民健身活动，办好奥运火炬南通境内传递活动和市第九届运动会。实施有线电视数字化整体转换，力争市区转换25万户。继续推进农村有线广播电视共缆传输双入户，力争入户比例超过60%。积极繁荣文艺创作，力争参评全国"五个一"工程奖实现新突破。加快文化产业发展。发展壮大广播电视、印刷出版、文艺演出等传统产业；加快发展文博、工艺美术、民族民间工艺等特色产业；大力扶持发展文化创意、动漫游戏等新兴产业。

　　（八）进一步保障人民权益，努力实现民主政治新发展。大力发扬社会民主。不断健全民主形式，保证人民依法实行民主选举、民主决策、民主管理、民主监督。扩大基层民主，充分发挥城乡居（村）民自治等社会自治功能。自觉接受监督。自觉接受人大及其常委会的法律监督、工作监督。创造条件让人大代表享有重大事项知情权，在日常工作中广泛听取人大代表意见。严格执行人大及其常委会的决议、决定，虚心听取并认真落实审议意见，及时主动报告工作，规范性文件及时报人大常委会备案审查。自觉接受政协、民主党派的民主监督和社会各界的监督。积极支持政协履行职责和民主党派参政议政，重大事项进行民主协商。高度重视办好人大议案、政协建议案和人大代表建议、政协委员提案。进一步做好民族、宗教和侨务、对台工作。深入推进法治南通建设。进一步落实《法治南通建设实施纲要》，继续开展法治合格县（市、区）、合格乡镇（街道）创建活动，深入推进"五五"普法，引导群众依法参与公共管理，全面提高全民法治观念和法律素质。

　　各位代表，面对贯彻落实科学发展观的新要求，必须坚持执政为民，严格依法行政，努力建设"行为规范、公正透明、勤政高效、清正廉洁"的政府。加快建设服务型政府。继续争创全省最佳办事环境，全力化解好项目用地、企业融资、征地拆迁等发展难题。创新服务手段，提高服务效能，实施电子政务二期工程，加快"数字南通"建设。深入推进政务公开，完善各类公开办事制度，加强新闻发言人制度建设，保障人民群众的知情权、参与权、表达权、监督权。切实提高行政执行力。进一步完善目标管理责任制、行政首长负责制、行政问责制和责任追究制，确保责任明确、政令畅通。进一步加强调查研究，完善决策信息和智力支持系统，健全公众参与、专家论证和政府决定相结合的行政决策机制。狠抓各项工作落实，继续实行重大项目会办、重点工作讲评等一系列措施，着力提高落实成效。不断增强政府公信力。认真落实《全面推进依法行政实施纲要》，严格按照法定权限和程序履行职责。进一步健全行政执法责任制。深入开展纠风治乱和专项治理工作，坚决纠正损害群众利益的行为。坚持标本兼治、综合治理、惩防并举、注重预防的方针，更加重视制度建设和源头防腐，以反腐倡廉的实际成果取信于民。加大执法监察、廉政监察、效能监察和财政资金使用监管力度，强化重点领域、重点部门和重点项目的审计。厉行节约，勤俭办事，努力降低行政成本。着力提高公务员队伍素质。加强公务员队伍建设，特别是各级领导干部的素质和能力建设，教育公务员在服从大局、遵守公德等方面做好表率，努力建设一支忠于职守、作风过硬、形象优良、清正廉洁的公务员队伍。

　　各位代表，南通的发展前景无限美好，历史机遇催人奋进。让我们紧密团结在以胡锦涛同志为总书记的党中央周围，高举中国特色社会主义伟大旗帜，沿着党的十七大指引的方向，深入贯彻落实科学发展观，在省委、省政府和市委的正确领导下，紧紧依靠全市人民，开拓创新，再接再厉，为"全面达小康、建设新南通"而努力奋斗！
